# Predictors for Confidence in Self-Management of Falls and the Role of Fear of Falls

**DOI:** 10.1093/geroni/igaa057.964

**Published:** 2020-12-16

**Authors:** Qiwei Li, Becky Knight

**Affiliations:** University of North Texas, Denton, Texas, United States

## Abstract

Falls have been a crucial threat for older adults to stay independent. Once they have fallen, older adults are more likely to receive injuries and become people with disabilities. Conventionally, the measurement of fall efficacy focused on the capacity of performing certain activities such as walking or bathing without a fall. However, given the fact that one out of five older adults fall every year, self-efficacy in self-protection when falls do happen calls for a better understanding of confidence in self-management of a fall. Among predictors for fall prevention outcomes, “fear of falls” has received attention. However, “fear of falls” was largely missing in studies exploring self-management of falls in scenarios where falls do happen. This study explores the predictors for CSMoF including “fear of falls”. A series of simultaneous and hierarchical regression analyses with related interaction analyses and a path model were applied to determine the contribution of each predictor variable and the mediating role of “fear of falls”. The findings of the study reported that demographic characteristics, chronic conditions, and perceptions of falls were associated with CSMoF. The path analysis confirmed the mediating role of “fear of falls” as the indirect effects were occupying substantial percentages in the total identified effects. “Fear of falls” should continue to be a core of fall prevention programs and is particularly important for programs that aim to teach older adults what to do when they fall, whom to call for help, and how to avoid injuries upon falling.

